# Hypermutation takes the driver’s seat

**DOI:** 10.1186/s13073-015-0159-x

**Published:** 2015-03-28

**Authors:** Matthias Schlesner, Roland Eils

**Affiliations:** Division of Theoretical Bioinformatics (B080), German Cancer Research Center (DKFZ), Heidelberg, 69120 Germany; Department for Bioinformatics and Functional Genomics, Institute for Pharmacy and Molecular Biotechnology (IPMB) and BioQuant, Heidelberg University, Heidelberg, 69120 Germany; Heidelberg Center for Personalised Oncology (DKFZ-HIPO), German Cancer Research Center (DKFZ), Heidelberg, 69120 Germany

## Abstract

Most pediatric tumors have only very few somatic mutations. However, a recent study revealed that a subset of tumors from children with congenital biallelic deficiency of DNA mismatch repair exhibits a mutational load surpassing almost all other cancers. In these ultra-hypermutated tumors, somatic mutations in the proofreading DNA polymerases complement the congenital mismatch repair deficiency to completely abolish replication repair, thereby driving tumor development. These findings open several possibilities for exploiting ultra-hypermutation for cancer therapy.

## Cancer mutational load varies over several orders of magnitude

Whole genome sequencing of hundreds to thousands of tumors from several cancer types has drastically increased our understanding of mutated cancer genomes. The mutational load of tumors from different cancer types varies over several orders of magnitude. At one extreme are most childhood cancers, with pediatric mixed-lineage leukemia-rearranged acute lymphoblastic leukemia having the lowest numbers observed yet in any cancer, with a median of only 111 somatic single-base mutations (SSMs) genome-wide [1]. At the other extreme are many hypermutated cancers, such as lung cancers or cutaneous melanoma, with hundreds of thousands of SSMs [2].

Hypermutation in cancer can be caused by exposure to exogenous mutagens (for example, UV light in melanoma or tobacco smoke in lung cancer) or endogenous mutagenic processes (for example, activation-induced deaminase in chronic lymphocytic leukemia and B-cell lymphomas and excessive APOBEC activity in multiple cancers) [3]. Alternatively, hypermutation can also be a consequence of defects in DNA repair mechanisms that normally ensure replication fidelity. In particular, two mechanisms limit the error rate during DNA replication to roughly one to five errors per cell division. Firstly, the proofreading ability of DNA polymerase δ and ε efficiently corrects most misincorporation events directly during DNA synthesis. Secondly, the DNA mismatch repair (MMR) system recognizes and corrects the remaining errors that arise during replication and also repairs several forms of DNA damage, thereby preventing the manifestation of mutations in the next round of replication. A third form of hypermutation affecting the inactive X chromosome seems to be present in virtually all cancers from female patients and shows no correlation to specific mutational signatures or DNA repair defects [4].

In a recent study, Adam Shlien and colleagues investigated tumors from children with congenital biallelic mismatch repair deficiency (bMMRD) by whole genome and exome sequencing [5]. Strikingly, all malignant brain tumors from bMMRD patients showed extremely large numbers of SSMs with an average of 249 SSMs per megabase. This mutation rate exceeds the average mutation rate even of highly mutated adult cancers by one order of magnitude; thus, they are called ‘ultra-hypermutated’.

## Ultra-hypermutation requires a second hit on the DNA safeguards

A secondary mutation in one of the proofreading polymerases δ and ε has been observed in all ultra-hypermutated cancers. The combination of congenital bMMRD and somatic *PolD1*/*PolE* mutations abolishes both key mechanisms to guarantee replication fidelity. Non-neoplastic tissue from bMMRD patients (which lacks *PolD1*/*E* mutations) did not show increased mutation numbers, indicating that the second hit is a prerequisite for ultra-hypermutation. bMMRD alone seems to lead to slow accumulation of mutations over time, resulting in moderately increased mutational load in bMMRD tumors lacking polymerase mutations, compared with MMR-intact tumors of the same tissue. Unless a second mutation in *PolD1*/*PolE* opens the door for ultra-hypermutation, it can take years until a sufficient number of drivers is acquired. Interestingly, mutations in either *PolD1* or *PolE* give rise to ultra-hypermutation in bMMRD cells. However, while both bMMRD/*PolD1* and bMMRD/*PolE* tumors show similarly high numbers of SSMs, their dominating mutational signatures are markedly different. *PolE*-mutated tumors are dominated by C > A and T > G transversions in a TCT or TTT context, respectively, while *PolD1*-mutated tumors bear predominantly C > A transversions in a CCN context. Since the *PolD1* and *PolE* mutations affect the intrinsic proofreading activity, the observed mutational patterns could reflect the initial error signatures of the polymerases.

## Ultra-hypermutation as a driver of cancer progression

The impact of hypermutation on cancer initiation and progression is not always clear. Generally, a higher mutational load increases the probability that a cell acquires sufficient driver mutations to undergo malignant transformation. However, hypermutation in a fully developed tumor might also be a passenger effect resulting from DNA repair deficiency acquired during tumor evolution. The *PolD1* and *PolE* mutations in the bMMRD/polymerase tumors occurred early in the development of the tumors and affected highly conserved residues. Additionally, these tumors almost completely lacked DNA copy number variants, suggesting that bMMRD/polymerase tumors are virtually exclusively SSM-driven. This suggests that the complete breakdown of replication repair, resulting in an explosion of SSMs, is an early mechanism associated with tumor initiation and drives tumor progression.

In another recent study, Supek and Lehner have shown that the lower mutation rates observed in early replicating regions of cancer genomes are due to more effective MMR and not due to different initial mutation rates [6]. Virtually all genes fulfilling essential cellular functions are early replicating. Hence, enhanced MMR in early replicating regions might be a protective mechanism to prevent damage of essential cellular mechanisms. Hypermutation caused by deficient DNA repair is not suppressed in early replicating regions, thereby undermining this protection. Therefore, with a similar number of genome-wide mutations spread evenly across the genome, hypermutation caused by defective DNA repair could have a higher probability than hypermutation caused by mutagens to affect early replicating genes and thus result in a fatal combination of mutations leading to malignant transformation. This might explain why DNA repair deficiencies such as bMMRD lead to tumor development during childhood in virtually all patients, while cancers associated with mutagen exposure often arise only after decades.

## What are the implications for treatment?

It can be expected that ultra-hypermutated cancers, due to their enormous mutation rate, exhibit sufficient genomic flexibility to rapidly acquire resistance against most therapies and, in particular, targeted therapies. Indeed, since the high mutation rate results in enormous tumor heterogeneity, most likely already at the beginning of a therapy, the mutations conferring resistance to the chosen drug will be present in a subset of tumor cells. On the other hand, as suggested by Shlien and colleagues, repair deficiency and the high mutational load might be the Achilles’ heel of ultra-hypermutated tumors. There seems to be an upper boundary for the mutational load in cancer. The cells from bMMRD/polymerase cancers acquire up to 600 new mutations with each cell division. However, when 10,000 to 20,000 exonic mutations are reached, the tumors seem to hit an upper limit of the tolerable mutational load. Neither pediatric nor adult cancers with constitutional and somatic MMR/polymerase defects exceeded this mutation level [2,3,5]. Considering this upper boundary for the mutational load, the already huge number of mutations and the complete lack of replication repair should make ultra-hypermutated cancer cells highly sensitive to DNA-damaging agents. However, in pediatric patients in general, and in children with congenital MMR-deficiency in particular, these drugs have a very high risk of severe side effects, including an increased risk for secondary tumors.

Other strategies to exploit ultra-hypermutation for cancer therapy might be safer options. The upper boundary for the mutational load indicates that ultra-hypermutated tumor genomes are at a point where damage of additional genes confers a likely selective disadvantage because essential cellular functions are impaired. Therefore, ultra-hypermutated cancers might be good candidates for exploitation of passenger vulnerabilities as proposed by Aksoy, Sander and others [7]. This approach focuses on essential cellular functionalities, which can be carried out by multiple partner proteins - for example, isoenzymes. If the tumor cells lose all but one partner protein due to mutational inactivation, the remaining partner will be essential for tumor cells, but not for normal cells. Inhibition of the remaining partner will thus specifically hit tumor cells. Since this therapeutic strategy is based on functionalities that the tumor has lost (and which usually cannot be gained again through additional mutations), it can be expected to be more robust against the development of resistance mechanisms than other targeted therapies. However, the challenge in this strategy is to identify defects that occurred very early on in the tumor development, and is further complicated by the requirement for biallelic defects. Otherwise, the presence of tumor cells without the targeted defect will guarantee tumor growth and impede sustained response to therapy.

Other possible strategies to exploit are immunotherapies [8], given the high number of mutations that might render ultra-hypermutated cancers more easily targetable than other tumor types. Immunotherapies exploit the fact that cancer cells expose antigens not found on normal cells. Such tumor-specific antigens arise when mutations generate protein sequences normally not present in the human body. With several thousands of exonic mutations, ultra-hypermutated cancers should offer a wide repertoire of tumor-specific antigens and should thus be promising targets for a tumor-specific immune response.

Genome analysis of bMMRD tumors has inspired several new therapeutic options for children with bMMRD-associated cancer. Since mismatch repair deficiency (MMRD) is common in many cancers, studying bMMRD tumors might aid the understanding of how and when MMRD contributes to tumor progression. Finally, investigation of bMMRD tumors may provide general insights into cancer biology: with 10,000 to 20,000 exonic mutations per tumor, in a cohort of moderate size every gene will be affected by random mutations. Potentially deleterious mutations will not be observed only in those genes, where impaired function is a selective disadvantage for the cancer cells. Analysis of the significantly unmutated genes in a cohort of ultra-hypermutated tumors can thus reveal cellular functions that are essential for cancer cells.

## Abbreviations

bMMRD: Biallelic mismatch repair deficiency
MMR: Mismatch repair
MMRD: Mismatch repair deficiency
SSM: Somatic single-base mutation
